# After Pains

**Published:** 1907-08-31

**Authors:** 


### Gynæcology.

### AFTER PAINS.

Abdominal pain is not at all an uncommon symptom during the first two or three days after delivery. When the pain is continuous it is generally due to bruising of the abdominal parietes. In cases where it has been necessary to hold the uterus firmly and for some time after delivery it is quite easy to cause a certain amount of bruising of the peritoneum and other structures of the abdominal wall. Such a complication is particularly likely to arise in cases where the abdominal walls are resistant, and in cases of post-partum hæmorrhage, in which a good grasp of the uterus is half the battle. One may occasionally see the actual bruise on the abdominal wall a day or two after delivery, and anyone who has performed a moderate number of post-mortem examinations on women dying soon after confinement will probably have noticed small sub-peritoneal hæmorrhages over the lower portion of the anterior abdominal wall. The pain caused by such bruises is of a dull, aching nature, and is continuous. Any movement on the part of the patient tends to intensify the pain, but it can be relieved easily and quickly by applying warmth in the shape of a hot fomentation over the abdominal wall. Apart from actual bruising of the abdominal wall one may notice that the recti muscles seem to suffer from stiffness, and this also may be relieved by warmth.

Intestinal colic is another common source of pain. It is often accompanied by a rise of temperature, even as high as to 104° and some quickening of the pulse rate, and there are cases which present some abdominal rigidity like that of early peritonitis. Pressure with the warm hand on the abdomen enables one to make a diagnosis between the two immediately, since it relieves considerably the pain of colic, but intensifies that of peritonitis.

In addition to these common causes of abdominal pain we find a variety of pain known as after-pains. These are painful contractions of the uterus after the completion of labour. Now it must be remembered that the uterus is a hollow muscle like the heart, which spends its life in alternate periods of contraction and relaxation. For some hours, and even days, after delivery the contractions may be distinctly felt by holding the uterus in the hand. But in the normal case these post-partum contractions are not painful.

Clinically, after-pains are noticed more in multiparæ than in primiparæ. When a labour is long and the expulsive forces strong, as in primiparæ, they may be almost entirely absent. On the other hand, after an unusually rapid labour they may be severe and proportionately painful. It is said that primiparæ are particularly liable to suffer after-pains in cases where the uterus has been over-distended by hydramnios or by a very large child, but the author cannot bear this out from his own personal experience. The pains may last for the first twenty-four hours only, or in later pregnancies may be prolonged until the fourth day even, so that occasionally the patient learns to dread the after-pains more than the pains of actual labour. As a rule the pains are unaccompanied by any rise in the temperature or pulse rate; it is only in very severe cases that there may be some accompanying fever.

The cause of after-pains is generally held to be the retention inside the uterus of pieces of placenta or membrane, and the formation of blood clot. The contractions by which the uterus endeavours to expel these foreign bodies give rise to the after-pains. Now this explanation does not serve in all cases. Pieces of placenta left behind in the uterus generally decompose and give rise to sapræmia. One has often seen large pieces of membrane and large blood clots expelled by the uterus during the puerperium without any pain whatever, in fact, without the patient knowing anything about it. The prevalence of after-pains among multiparæ is said to be due to the fact that in them the retraction of the uterus is not so complete as it is among primiparæ, and that therefore clots form inside the uterus, and give rise to after-pains during their expulsion. If this were true, as a general explanation, one would expect to find that the lochia in multiparæ were more profuse, but it is not so, and moreover in many of the most severe cases of after-pains nothing at all is expelled from the uterus. Therefore one cannot accept feeble retraction as the true explanation of after-pains.

In searching for the explanation of any symptom pathological anatomy is the first line of attack. What difference is there between the uterus of a multipara and the uterus of a primipara? The main difference is found in the structure of the uterine wall, for, whereas in the primipara it is smooth and practically homogeneous on section, in the multipara we find more fibrous tissue, and the arteries are thickened, hard, and prominent upon the surface of the section. Having already referred to the similarity between the heart and the uterus, we cannot refrain from pointing out that in cases where the coronary arteries of the heart undergo a sclerosis similar to that undergone by the uterine arteries after many pregnancies, the heart is liable to suffer from the spasmodic pains known as angina pectoris. Amyl nitrite relieves both pains. The nervous factor is, however, practically an unknown quantity in the problem of after-pains. All we can say is that women who suffer from spasmodic dysmenorrhœa are almost certain to have after-pains after delivery.

The treatment of after-pains is more successful if one works with the idea that after-pains are a species of cramp of the uterus. In a very few cases where retained blood clot is at the bottom of the trouble, ergot and hot douches may be successful. Hot douches are always useful in cases of bad after-pains, since the warmth acts as an antispasmodic. Drugs which relieve spasm are much the best to use in these cases. The specific for after-pains is antipyrin in ten-grain doses. The action of this drug is so efficacious in cases of after-pains that no other need be mentioned. It may be taken in cachets or in solution combined with a little spirits of chloroform and water. At the same time it is wise to relieve the bowels, and to place a hot-water bottle over the uterus.

